# Parapharyngeal space hemangiopericytoma treated with surgery and postoperative radiation- a case report

**DOI:** 10.1186/1758-3284-4-10

**Published:** 2012-04-05

**Authors:** Muhammad Mohsin Fareed, Abdullah Suleiman Mazaed Al Amro, Rashad Akasha, Mansour Al Assiry, Mushabbab Al Asiri, Mutahir Tonio, Yasser Bayoumi

**Affiliations:** 1 Department of Radiation Oncology, King Fahad Medical City, Riyadh 11525, Saudi Arabia; 2 Department of E.N.T, King Fahad Medical City, Riyadh 11525, Saudi Arabia

**Keywords:** Hemangiopericytoma, Surgical resection, Postoperative radiation, Parapharyngeal space

## Abstract

Hemangiopericytoma (HPC) is a rare tumor of uncertain malignant potential arising from mesenchymal cells with pericytic differentiation. It accounts for 3-5% of soft tissue sarcomas and 1% of vascular tumors. It usually presents in 5^th ^to 6^th ^decade of life. Most common sites are limbs, pelvis and head and neck. About 20% of all hemangiopericytomas are seen in head and neck, mostly in adults. Usually it presents in orbit, nasal cavity, oral cavity, jaw, parotid gland, parapharyngeal space, masticator space and jugular foramen. Long term follow up is important because of imprecise nature of the histological criteria for prediction of biologic behavior.

We report herein a case of HPC in 66-year-old man, who presented in our department with headache, nasal obstruction and dysphagia. A neck computer tomography scan and magnetic resonance imaging showed a large left parapharyngeal mass bulging into nasopharynx and oropharynx with extension to pharyngeal mucosal surface and causing narrowing of airways and total obstruction of left posterior nostril. Angiography showed a highly vascular neoplasm. Initially he was managed as a case of schwannoma and embolization was done but with no response. An attempt to do complete surgical resection was made, but due to its critical position, it was not possible. During surgery, highly vascularised tumor was found. The histopathologic examination revealed a vascular tumor consistent with hemangiopericytoma G-II. The patient had normal postoperative course of healing and was given adjuvant radiation. He is on regular follow up without signs of recurrence or metastases.

In summary, parapharyngeal space is a rare site of presentation for hemangiopericytoma which is highly vascular tumor, requiring extensive work up including magnetic resonance imaging, computed tomography scan and angiography. Complete surgical excision should be attempted. Postoperative radiation is indicated in cases of incomplete resection.

## Background

Hemangiopericytoma is a rare tumor type. It originates in a specific cell type called pericytes, identified by Rouget in 1873 and subsequently described by Zimmermann in 1923. Just above 300 cases of HPC have been reported since Stout and Murray described HPCs as "vascular tumors arising from Zimmerman's pericytes" in 1942. These cells are arranged alongside capillary vessels and have smooth muscle characteristics. They are responsible for vessel caliber regulation owing to their contractile capability, modulating both flux and permeability [1-3]. Regarding etiology, a past history of trauma, prolonged steroid use and hypertension are said to have some correlation, but such correlations have not been formally demonstrated [4].

Hemangiopericytoma constitutes about 3-5% of all soft tissue sarcomas and about 1% of all vascular tumors. The head and neck incidence is 15-30% and it is mostly seen in adults. In the head and neck region it is usually seen in the orbit, nasal cavity, oral cavity, jaw, parotid gland, parapharyngeal space, masticator space and jugular foramen [5,6]. The patient described in our report presented with symptoms of parapharyngeal compression due to such a tumor. About 12 cases of parapharyngeal space hemangiopericytomas have been previously reported. Diagnosis is made histologically, but it is difficult to predict the behavior of the tumor in an individual patient [7]. It has low potential for local recurrence or metastasis. The treatment of choice is total local excision. Adjuvant radiotherapy and chemotherapy may be employed and, although the literature is not quite clear about their results, recent studies have suggested that their use is indicated mostly in cases where only partial resection was performed. Patients with hemangiopericytoma should be regularly monitored for local recurrence and systemic tumor spread [8]. We present a case of left parapharyngeal hemangiopericytoma treated with surgical resection followed by adjuvant radiotherapy.

## Case presentation

**Figure 1:**
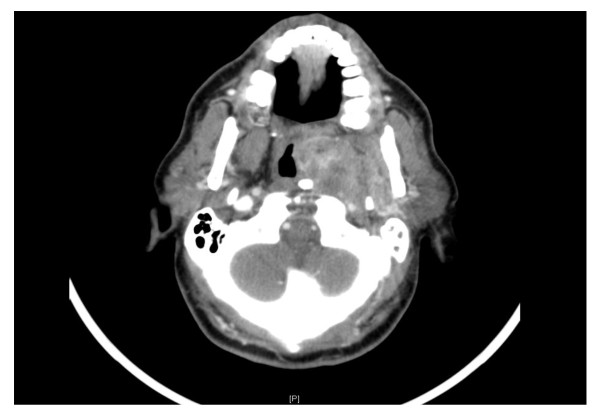
**CT scan showing left parapharyngeal soft tissue mass protruding into oro and nasopharynx**.

**Figure 2:**
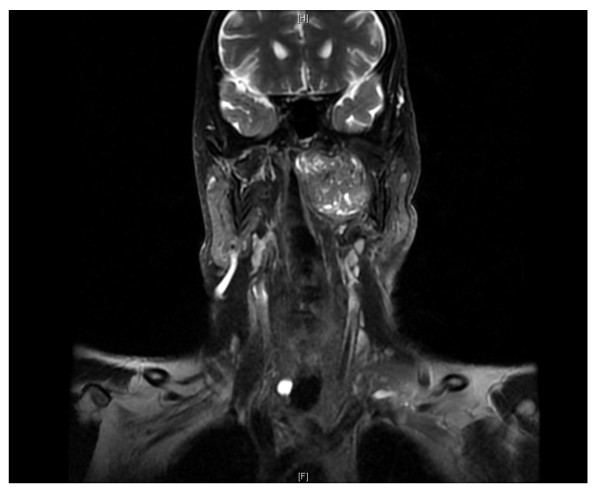
**MRI showing 5 × 6 × 3.3 cm enhancing left parapharyngeal mass compressing the upper internal jugular vein**.

A 66-year old male with known diagnosis of diabetes mellitus, hypertension, cerebrovascular accident and benign prostatic hyperplasia presented in December 2009 with 3 months history of nasal obstruction, headache and difficulty in swallowing. For the past 1 month, he had developed numbness over the right side of face. CT scan head and neck on 27/12/2009 showed well defined heterogeneously enhancing 3.6 × 6.6 × 5.5 cm left parapharyngeal solid mass with multiple small cystic components, bulging into nasopharynx and oropharynx with extension to pharyngeal mucosal surface (Figure 1). The mass was causing narrowing of airways, total obstruction of left posterior nostril, remodeling of medial and lateral left pterygoid plates and abutting the left internal carotid artery. There was left maxillary sinus retention cyst along with opacification of left middle air and mastoid air cells. D/D of schwannoma vs. glomus vegale was made and MRI suggested. MRI neck done on 30/12/2009 confirmed the left parapharyngeal pear shaped mass occupying the pre-styloid parapharyngeal space extending to post-styloid space bulging into the oro and nasopharynx, likely representing schwannoma(Figure 2). Carotid and vertebral arteries duplex US on 9/1/2010 showed patent bilateral common, external and internal carotid arteries along with patent vertebral arteries. There was 1.8 mm intimal wall thickening at left CCA. External carotid angiogram and embolization of left parapharyngeal tumor was done on 10/1/2010. Enlargement of left external carotid artery caliber along with enlargement of ascending pharyngeal artery mainly supplying left parapharyngeal tumor was seen. There was no significant shunting to the intracranial circulation. Left parapharyngeal tumor, likely schwannoma was embolized using 25% Glubran with adequate devascularization. Patient tolerated the procedure without any complications.

He remained on regular follow up until his CT scan head and neck on 19/1/2011 demonstrated interval progression with increase in the size of mass lesion occupying the prestyloid compartment of left parapharyngeal space to 6.5 × 7.5 × 3.5 cm anteriorly abutting the nasal choana. It was now involving the deep lobe of left parotid gland. It was causing mild narrowing of the airways. The upper part of left internal jugular vein was not visualized at the level of jugular foramen.

**Figure 3:**
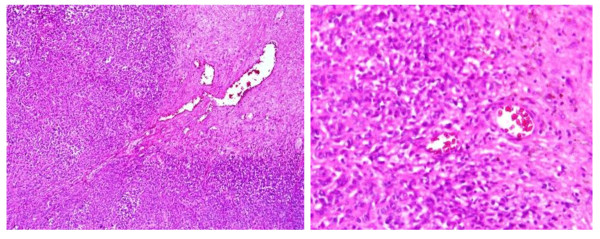
**Vascular neoplasm with sheets of pericytes in between thin wall blood vessels consistent with hemangiopericytoma**.

He underwent surgical excision of left parapharyngeal mass along with left parotidectomy and left level II neck node dissection on 1/5/2011. The tumor consisted of multiple small and large variegated gray white to tan firm pieces of tissue measuring in aggregate 8 × 7 × 1.5 cm. Histopathology showed vascular neoplasm consisted of sheets and groups of spindle and oval shaped cells (pericytes) in between thin wall blood vessels in some areas with stag horn pattern consistent with hemangiopericytoma G II (Figure 3). Left Parotid mass and two left cervical level II lymph nodes showed no significant pathologic changes.

He was seen in radiation oncology clinic on 08/02/2012. Examination revealed ECOG PS O with left facial nerve palsy. There was well healed scar mark over the left upper neck. After discussing the case in tumor board, it was decided to give him postoperative radiation therapy. He received 60 Gy in 30 fractions, 200 cGy/day, 5 days per week by IMRT technique to tumor bed which started on 2/6/2011 and completed on 2/8/2011. He tolerated radiation treatment quite well developing only G-1 skin reaction in last week of radiation therapy. He is on regular follow up till now and is disease free. His CT scan neck done on 27/11/2011 showed no evidence of recurrence or residual disease.

## Discussion

Hemangiopericytoma is uncommon in the head and neck. Stout and Murray described 691 cases of vascular tumors (1942), and only nine of these were hemangiopericytoma. The most frequent anatomic sites for HPC are the extremities, the pelvis, and the head and neck, accounting for 80% of the total cases [9]. Its incidence in the head and neck is less than 20% mostly affecting soft tissue around the oral cavity, sinus tract and meninges and rarely, it affects the orbit, parotid gland, skull base and temporal bone. Hemangiopericytoma affecting parapharyngeal region is even more uncommon. Its peak prevalence is in the sixth to seventh decade of life with no sex predilection. The etiology is unknown, although it has been linked to hypertension, trauma, prolonged steroid use and hormonal imbalance [10]. Our patient is hypertensive but has no history of trauma or prolonged steroid use.

Differential diagnosis of hemangiopericytoma includes juvenile hemangioma, glomus tumor, angiosarcoma, leiomyoma, leiomyosarcoma, schwannoma, mesothelioma, liposarcoma, benign and malignant histiocytoma, synovial sarcoma, chondrosarcoma, neuroblastoma, adenoid cystic carcinoma and mixed cell tumor. Angiographic features may help in differentiating hemangiopericytoma from other hyper vascular lesions. Tomography, radiography and angiography are not specific. Radiographically, the tumor consists of well-circumscribed, radiopaque soft tissue mass often displacing surrounding structures [11].

Characteristically, HPC is a well-circumscribed, brown, spongiform lesion, surrounded by a pseudo-capsule, often with small satellite nodules separate from the main tumor mass [9]. They can be lobulated or nodular, firmly attached to muscle or fascia, and soft, firm or friable. They usually consist of small closely packed cells with ill defined cytoplasm and darkly stained nuclei microscopically. Slit like or sinusoidal vascular spaces are interspersed between cells. Vimentin is the only marker expressed consistently in hemangiopericytoma [11]. Enzinger reported the following characteristics that are compatible with a high-grade tumor: nuclear atypia, necrosis, hemangioma, presence of more than four mitosis per microscope field and size greater than 6.5 cm [12]. However, Stout and Murray did not notice any correlation between the mitosis level and tumor behavior. They noted that the ten-year survival rates of patients with lesions that presented fewer than four mitosis per microscope field, absence of necrosis and size below 6.5 cm were respectively 77%, 81% and 92%. On the other hand, when the tumor presented more than four mitosis per field, necrosis and size greater than 6.5 cm, the ten-year survival rates were respectively 9%, 29% and 63%. Recurrence indicates a poor prognosis and many such cases develop distant metastasis. Studies have shown metastasis rates ranging from 18% to 69%.

Plain radiography is nonspecific, however angiography is recommended for confirmation of diagnosis of vascular neoplasm, preoperative definition of blood supply, and embolization of tumor. Characteristic features on angiography include hypervascularity, radially arranged branching vessels around and inside the tumor, and longstanding, well-demarcated tumor stains. MRI provides useful preoperative information about the extent of the tumor and its relationship to the surrounding structures and distinguishes between intraparotid and extra-parotid neoplasms. Computerized tomography is helpful in the evaluation of bony erosions [13]. The pre-operative evaluation of a hemangiopericytoma must include a thorough imaging evaluation with computerized tomography and magnetic resonance imaging, even if results may not be specific for hemangiopericytoma. Angiography and pre-operative embolization may be performed in cases of large tumors with significant vascularity [14].

The treatment of choice is primary resection with a wide safety margin. Patients undergoing complete resection showed a 100% median survival at 60 months [9]. The usefulness of adjuvant radiation therapy has not been fully supported in the literature, its primary role is confined to incomplete resection. It can also be used in cases of local recurrence, metastases and inoperable tumors [15,16]. Since recurrence and metastasis can occur after many years (up to 7 years in some series) a lifelong regular follow-up is necessary [17].

## Conclusions

Parapharyngeal space hemangiopericytoma is a rare entity found in a rare site. High index of suspicion is required to diagnose these cases. Extensive radiological as well as histological investigations need to be carried out. An attempt should be made for complete surgical resection. Postoperative radiation is indicated in cases of incomplete resection.

## Consent

Written informed consent was obtained from the patient for publication of this Case report and any accompanying images. A copy of the written consent is available for review by the Editor-in-Chief of this journal.

## Abbreviations

HPC: Hemangiopericytoma; MRI: Magnetic resonance imaging; CT: Computed tomography; CCA: Common carotid artery; D/D: Differential diagnosis; ECOG: Eastern Cooperative Oncology Group; IMRT: Intensity modulated radiation therapy

## Competing interests

The authors declare that they have no competing interests.

## Authors' contributions

MMF collaborated between treating surgeon and primary radiation oncologist, collected the data, radiology and pathology slides and wrote manuscript, AAA was the primary radiation oncologist who planned for postoperative radiation, MAA was the primary surgeon who did the maximam possible surgery, YB assisted in radiation oncology treatment planning and execution, MT and RA helped in writing manuscript, while MAA provided valuable guidance in formatting and revising the manuscript. All authors read and approved the final manuscript.
